# Buffering Mechanisms in Aging: A Systems Approach Toward Uncovering the Genetic Component of Aging

**DOI:** 10.1371/journal.pcbi.0030170

**Published:** 2007-08-31

**Authors:** Aviv Bergman, Gil Atzmon, Kenny Ye, Thomas MacCarthy, Nir Barzilai

**Affiliations:** 1 Department of Pathology, Albert Einstein College of Medicine, New York, New York, United States of America; 2 Department of Molecular Genetics, Albert Einstein College of Medicine, New York, New York, United States of America; 3 Department of Neuroscience, Albert Einstein College of Medicine, New York, New York, United States of America; 4 Institute for Aging Research Department of Medicine, Albert Einstein College of Medicine, New York, New York, United States of America; 5 Department of Epidemiology and Population Health, Albert Einstein College of Medicine, New York, New York, United States of America; University of California Los Angeles, United States of America

## Abstract

An unrealized potential to understand the genetic basis of aging in humans, is to consider the immense survival advantage of the rare individuals who live 100 years or more. The Longevity Gene Study was initiated in 1998 at the Albert Einstein College of Medicine to investigate longevity genes in a selected population: the “oldest old” Ashkenazi Jews, 95 years of age and older, and their children. The study proved the principle that some of these subjects are endowed with longevity-promoting genotypes. Here we reason that some of the favorable genotypes act as mechanisms that buffer the deleterious effect of age-related disease genes. As a result, the frequency of deleterious genotypes may increase among individuals with extreme lifespan because their protective genotype allows disease-related genes to accumulate. Thus, studies of genotypic frequencies among different age groups can elucidate the genetic determinants and pathways responsible for longevity. Borrowing from evolutionary theory, we present arguments regarding the differential survival via buffering mechanisms and their target age-related disease genes in searching for aging and longevity genes. Using more than 1,200 subjects between the sixth and eleventh decades of life (at least 140 subjects in each group), we corroborate our hypotheses experimentally. We study 66 common allelic site polymorphism in 36 candidate genes on the basis of their phenotype. Among them we have identified a candidate-buffering mechanism and its candidate age-related disease gene target. Previously, the beneficial effect of an advantageous *cholesteryl ester transfer protein* (CETP-VV) genotype on lipoprotein particle size in association with decreased metabolic and cardiovascular diseases, as well as with better cognitive function, have been demonstrated. We report an additional advantageous effect of the CETP-VV (favorable) genotype in neutralizing the deleterious effects of the *lipoprotein*(*a*) (LPA) gene*.* Finally, using literature-based interaction discovery methods, we use the set of longevity genes, buffering genes, and their age-related target disease genes to construct the underlying subnetwork of interacting genes that is expected to be responsible for longevity. Genome wide, high-throughput hypothesis-free analyses are currently being utilized to elucidate unknown genetic pathways in many model organisms, linking observed phenotypes to their underlying genetic mechanisms. The longevity phenotype and its genetic mechanisms, such as our buffering hypothesis, are similar; thus, the experimental corroboration of our hypothesis provides a proof of concept for the utility of high-throughput methods for elucidating such mechanisms. It also provides a framework for developing strategies to prevent some age-related diseases by intervention at the appropriate level.

## Author Summary

Previous research showed that the frequency of deleterious genotype of some age-related disease decreases its prevalence as the population ages, as expected, since subjects with deleterious genotype are weeded out due to mortality. There exists, however, a set of age-related genes whose deleterious genotype indeed decreases up to ages 80–85, but subsequently increases monotonically, until by age 100 its prevalence is similar to that at age ∼60. Why is a known harmful genotype so prevalent among centenarians? Most likely because this genotype is protected by longevity genes. We corroborated this hypothesis by studying gene–gene interactions between age-related disease genotypes and longevity genotypes. Our findings suggest that individuals with the favorable longevity genotype can have just as many deleterious aging genotypes as the rest of the population because their longevity genotype protects them from the harmful effects of the other. We identify genes contributing to extreme lifespan as well as their counterpart, age-related disease genes. Our findings provide a proof of concept for the utility of high-throughput methods, and for elucidating mechanisms by which longevity genes buffer the effects of disease genes. Our approach gives hope for developing new medications that will protect against several age-related diseases.

## Introduction

While life-style factors such as obesity have been identified as limiting factors in life expectancy [1], genetic factors have been implicated in, and are considered central to, the process of aging. These genetic factors are obvious simply from observing the change in maximal life span between species, and the effect of single genes on longevity in several invertebrates [2–4]. However, the long list of biological processes associated with aging has not permitted all the causes of mammalian aging to be determined. Monogenetic disorders such as abnormalities in the LDL receptor gene [5,6], the rare helicase gene defects in Werner syndrome that prevent subjects from attaining normal lifespan [7], and the more common BRCA1 and BRCA2 genes in breast cancer [8], all represent genetically determined phenotypic defects associated with early mortality. However, these are not necessarily common mechanisms of aging, and despite the evidence for a substantial genetic component, the inherited biological factors [9] that define lifespan in long-lived humans remain unknown. In population-based studies, the incidence and prevalence of age-related disease can be a marker of aging. For example, in our Ashkenazi Jewish study, the prevalence of homozygosity for the −641C allele in the APOC-3 (apolipoprotein C3) was found to be significantly higher among centenarians and their offspring than in the control [10]. Significantly lower serum levels for APOC-3 and unique lipoprotein phenotype (lipoprotein particle size) were noted among carriers of the −641C homozygote [10]. In previous work, we examined the effect of the genotype on aging by monitoring age–related disease as a clinically relevant surrogate for aging. We showed that this favorable APOC-3 genotype was associated with lower prevalence of hypertension, CVD, and metabolic syndrome, in addition to being significantly more prevalent among centenarians and their offspring than that of the control group. Furthermore, a strong correlation was found with increase homozygosity for the 405V variant in CETP (cholesteryl ester transfer protein) [11] in centenarians, also associated with a unique lipoprotein phenotype and lower prevalence of metabolic syndrome and with preservation of cognitive function [12]. These observations further confirm that aging is a complex trait, and additionally, that genes associated with aging often have pleiotropic effects. To address the complexity of aging, we adopted an evolutionary approach in searching for genes associated with longevity and the “aging phenotype,” which includes age-related diseases, in humans. Evolutionary theory can be summarized as the study of how genetic variation within a population is translated into variation between populations in response to natural selection, *i.e., differential reproduction* over the course of many generations. Similar principles can be applied to the study of changes in the genetic makeup of populations in response to *differential survival* over the course of one or two overlapping generations. Differential survival in response to mortality will therefore be reflected in the prevalence of genotypes underlying the process of aging and longevity.

The studies reported here are based on the following three assumptions: first, exceptional longevity is a rare phenotype; second, genotypes associated with age-related diseases are “weeded out,” while those genotypes associated with survival, longevity genes, are enriched in an advanced age subpopulation; third, some favorable longevity genotypes may act to buffer the deleterious effects of other genes that lead to age-related diseases. It is this third assumption that makes it possible to discriminate between age-related disease genotypes and the longevity genotype. Given cohorts representing each decade of the lifespan, one can examine whether those who continue to survive exhibit biologically distinctive phenotypes and genotypes, when compared with those of younger cohorts. Thus, the relative prevalence of favorable longevity genotypes within the population can be expected to rise monotonically rather than abruptly or intermittently over the life course; conversely, the prevalence of age-related diseases genotype is expected to monotonically decrease; however, the prevalence of those deleterious genotypes that are buffered may, paradoxically, be found to remain the same (or increased) among individuals with extreme lifespan. Using populations of Ashkenazi Jews consisting of individuals in the age range 50–110 years (including ∼400 individuals between ages 95–110), indeed we observe a monotonic increase with age for three genotypes: 1) the CETP gene codon 405 isoleucine to valine variant (CETP *VV*); 2) the APOC-3 gene codon A (−641) C variant (APOC-3 *CC*); 3) a deletion at +2019 in the adiponectin (*ADIPOQ*) gene. The enrichment of the CETP genotype is supported by evidence from two independent populations [11,13]. Finally, we may explain why the CETP-VV genotype appears to exhibit an additional advantageous effect—the neutralization of the deleterious effects of the LPA gene: from this follows the observed high prevalence of the deleterious genotypic variant of LPA among the centenarians.

### Rationale for Genotyping Centenarians and Expected Pattern with Aging

**Figure 1:**
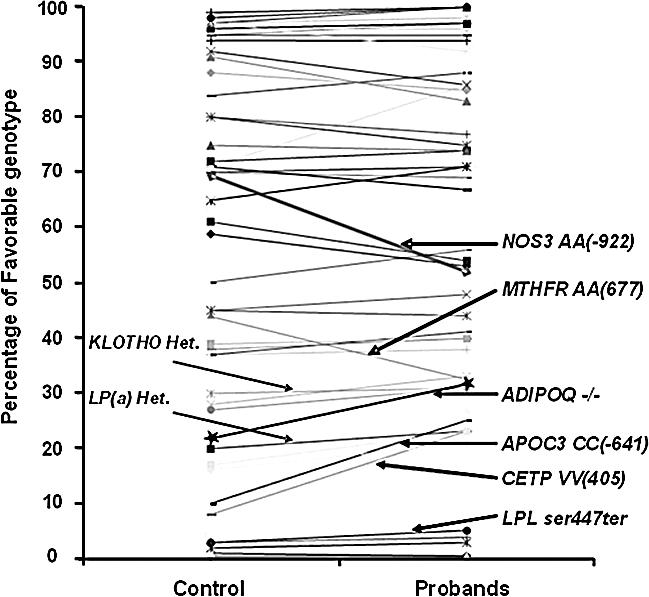
Genotypic Frequency Comparison between Control and Proband Genotypic frequencies of SNPs associated with some of the genes implicated in CVD. Comparison is between control individuals (∼70 years old), and probands (∼100 years old). Offspring of centenarians are excluded from this analysis. Significant change, *p* < 0.0066 (after applying Bonferroni correction), was found for two genes, CETP and APOC-3. Most genotypes exhibit no change in frequency between the two groups though they may still be factors contributing to lifespan.

**Figure 2:**
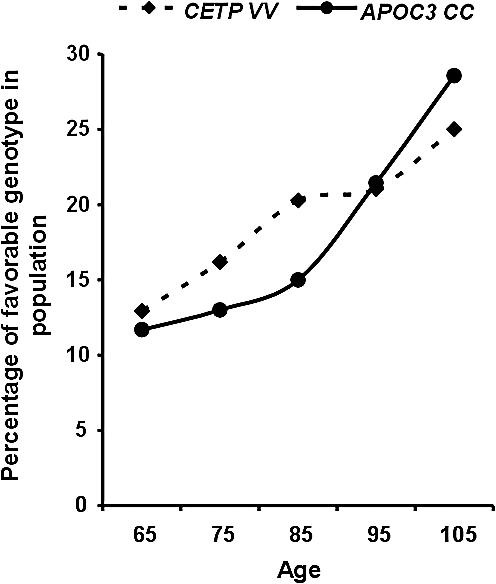
Frequency Trend of Two Buffering Genes Longevity genes are expected to exhibit monotonic increase in their favorable genotype when sampled in progressively older age groups. The graph shows a highly significant (*p* < 0.0006) monotonic increased frequency across ages for favorable genotypes in APOC-3 *CC,* and a significant (*p* < 0.047) for CETP-VV, fulfilling our definition for candidate longevity genes.

Aging is associated with a decline in the frequency of survivors attaining older ages; i.e., the frequency of centenarians in human populations is only ∼1/10,000 persons. Given the evidence of a genetic basis for longevity [14–16], we would expect the prevalence of favorable genotypes in genes contributing to prolonged lifespan—i.e., *longevity genes—*to be significantly higher among centenarians relative to their prevalence in a younger control population, as can be observed for the favorable genotypes of CETP-VV and APOC-3 in Figure 1 (see the discussion below). Furthermore, although at birth the probability of living more than 100 years is ∼1/10,000, this probability increases to ∼1/250 when an individual reaches his or her life expectancy (∼80 years old). We would therefore expect the frequencies of longevity genotype to increase monotonically in progressively older age groups. Figure 2 shows the expected monotonic increase for the same genotypes, CETP-VV and APOC-3 (see also Figure S1). In contrast, deleterious genotypes associated with “aging phenotype” (e.g., age-related *disease genes*) would be expected to decrease monotonically as mortality selects out individuals with these deleterious genotypes. These considerations suggest that changes in genotypic frequencies affecting lifespan in different age groups can be detected and used to determine the relevant genes associated with the aging process.

However, the two components, longevity and an aging phenotype, cannot be disentangled, since an increase of a favorable genotype in a longevity gene necessarily results in a decrease of the frequency of its complementary genotypes. Similarly, a decline in prevalence of a deleterious genotype at an age-related disease gene necessarily implies an increase in its non-deleterious alternatives. Thus, a simple analysis of monotonic increase or decrease in genotypic prevalence across age groups is not sufficient to discriminate between longevity genes and their counterpart age-related disease genes.

### Identification and the Interpretation of a *U*-Shape Pattern of Genotype with Aging

**Figure 3:**
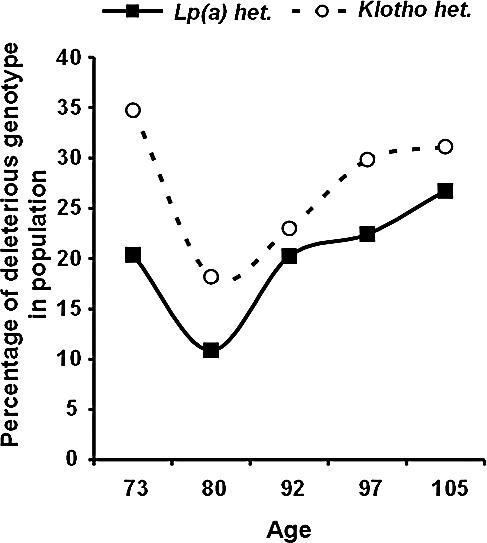
Observed *U*-Shape Trend of Age-Related Buffered Disease Genes Longevity genes are hypothesized to buffer the phenotypic effect of certain deleterious age-related disease genotypes, thus allowing the accumulation of the latter in a population endowed with longevity genotypes. Presented here are the frequency trends across ages of deleterious genotypes in KLOTHO and LPA. Frequencies decline until the population age is ∼80 years old, close to the current average lifespan, and then increases to nearly the frequency in younger age, fulfilling our definition of a buffered disease gene. We used a binomial model with identity link function with both linear and quadratic terms for age, and tested for the significance of the quadratic component. We found a statistically significant quadratic component at the level of *p* < 0.035 for both KLOTHO and LPA*.*

In this study, we introduce a possible solution to overcome this limitation. As a first step, we must consider the protective effect that longevity genes might confer and what effect this might have on lifespan. It has been shown that offspring of centenarians are healthier than their appropriate age-matched controls, supporting the notion of inheritance from centenarians to their offspring [10,12,16–20]. This observation lends support to the hypothesis that longevity genes might buffer the deleterious effect of certain genetically determined age-related diseases. Genes associated with the latter set of diseases are here termed *buffered disease genes.* Figure 3 shows the frequency trend of the deleterious genotypes of two age-related diseases genes, KLOTHO and LPA, and indeed, centenarians are endowed with significantly higher deleterious genotype than the elder control group (80 years old), and in the case of LPA, even than that of the younger group. The presence of a molecular buffering mechanism has already been discussed and observed in model organisms. Studies of buffering mechanisms such as the chaperone HSP90 have demonstrated that in the wild-type, under normal conditions, the hidden accumulated, mostly deleterious, genetic variation is buffered and not expressed phenotypically; however, when HSP90′s functionality is compromised, that same genetic variation is translated into, mostly deleterious, phenotypic variation [21,22]. Further theoretical analyses on the capacity to harbor and express such phenotypic variation have also been reported [23,24]. These theoretical findings have been corroborated by experimental data from model organisms [23,24] without any a priori assumptions regarding the potential genetic pathways. It is therefore reasonable to extend this approach to genetic hierarchies and pathways responsible for the process of aging in humans. We recognize that not all longevity genes act as buffering mechanisms. Our study therefore focuses on the discovery of those genes that have a buffering effect, and their targets, age-related disease genes. This equivalence suggests that the buffering effect longevity genes are hypothesized to possess, will allow the accumulation of deleterious allelic variants in buffered disease genes. In turn, we expect the prevalence of a deleterious age-related genotype among centenarians to be maintained at a level similar to that prior to the onset of the age-related disease within the (younger) control population. Centenarians, however, are rare in human populations; thus, an initial decline in the prevalence of the deleterious genotype in buffered disease genes is expected. As the population ages, the proportion of individuals endowed with favorable genotypes at longevity gene loci increases, as should the proportion of individuals carrying (the now buffered) deleterious age-related disease genotype. Thus, such deleterious genotypes should exhibit a *U*-shaped trend with progressive aging. The following more formal argument describes this more clearly. We first divide the population (at birth) into those protected by the longevity allele (group *P*) and those lacking it. We then further subdivide the latter group into those with deleterious allele in age-related disease gene (unprotected group *U*) and those with wild-type allele (group *N*). Since they are unprotected from the deleterious genes they are carrying, group *U* is expected to have the shortest mean lifespan, *d_U_*, of the three groups (*P*, *U*, and *N*). Assuming that the favorable genotype group *P* has the longest mean lifespan, *d_P_*, (*d*_P_ > *d*_N_ > *d*_U_), in part because the longevity allele confers a buffering effect against the deleterious alleles they may have, then we will observe a *U*-shaped curve, which is a consequence of the final population being dominated by group *P* (see Text S1 and Figure S3 for simulation results).

Figure 3 shows such a trend for the deleterious variant of two age-related disease genes, KLOTHO and LPA (see also Figures S1–S3).

### Gene–Gene Interactions To Identify Targets for Longevity Genes

**Figure 4:**
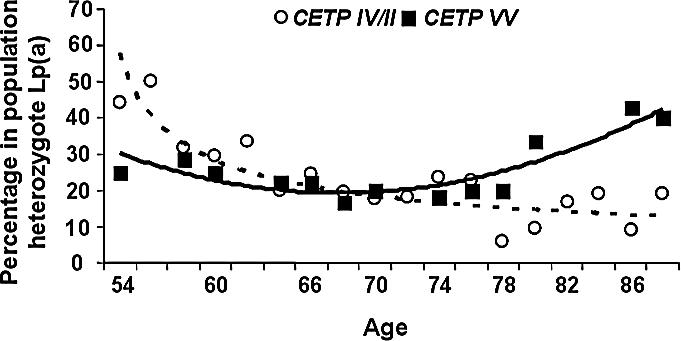
Interaction between CETP and the Buffered LPA Gene An interaction between the longevity gene and its target buffered disease gene is revealed by population subdivision. A subpopulation endowed with the favorable longevity genotype will show either no change, or an increase, in the frequency of its target deleterious genotype. In a population lacking the favorable longevity genotype, a monotonic decline (similar to the decline in nonbuffered disease gene) will be observed. Presented here are the frequency trends across ages of deleterious genotype in LPA in the subpopulation having favorable longevity genotype CETP-VV versus the subpopulation lacking it, i.e*.*, CETP*-IV* and CETP*-II*. The two trends show a significant difference (*p* < 0.037, see Text S1 for statistical considerations).

An important final step in our analysis is required—that is, to associate longevity genes with their potential targets—buffered disease genes. In the absence of favorable genotypes in longevity genes, the protective effect, and with it the accumulation of deleterious genotype in buffered disease genes, will not occur. Therefore, in a subpopulation lacking longevity-favorable genotypes, a monotonic decline in the prevalence of a deleterious genotype in a buffered disease gene is expected. In contrast, in a subpopulation possessing the favorable genotype, no change, or an increase, in the prevalence of deleterious buffered disease genes will be observed. Figure 4 shows such an interaction between the deleterious variant of LPA and the two variants of CETP (for further details see also Figure S2). A limiting factor in this final step is that, since centenarians are rare, we expect the favorable genotype in longevity buffering genes also to be rare among younger populations. To overcome this limitation, we make novel use of the centenarian offspring data. Inheritance assures that the genotypes among offspring will be enriched with favorable longevity genotypes. Thus, by admixing the offspring with a control population, we artificially enrich the prevalence of rare longevity genotypes. Because we only make use of the offspring population in this final step, the identification of potential longevity genes and buffered disease genes (and the analysis of their interactions) is not affected by the introduction of this artificial enrichment.

## Results

### Patterns of Genotypes with Aging

We present the analysis of the 66 common allelic site polymorphisms in 36 candidate genes for a lipoprotein phenotype we have studied (see Methods for the population and statistical considerations). Figure 1 visually represents the frequencies of their genotypes in ∼70 and ∼100-year-old subjects (see Table S1 and Text S1 for a complete list). Only the frequencies of homozygosity for the codon 405 valine (*V*) allele of CETP (*VV* genotype) and the homozygote *CC* genotype in the APOC-3 promoter region APOC-3 *C*(*-641*)*A*, have been determined as having a significantly greater prevalence among centenarians (both *p*-values from chi-square tests are less than 0.0001, and show statistical significance even after Bonferroni correction). Both genotypes have been previously associated with increased lipoprotein particle size, and thus are associated with a reduced risk for CVD. For that reason, we considered them favorable candidates for longevity genotypes [11,25].

The observed greater prevalence of these genotypes in centenarians compared with the control group as a whole does not, however, completely satisfy our first hypothesis. That is, for CETP-VV and APOC-3 *CC* to be considered favorable longevity genotypes, a *monotonic* increase with age is expected. Indeed, this turns out to be the case. Figure 2 shows a highly significant (*p* < 0.0006) monotonically increasing frequency trend for APOC-3 *CC*. For CETP, the monotonic increase trend is also found to be statistically significant (*p* < 0.047), making both APOC-3 *CC* and CETP longevity gene candidates. The statistical significance for a monotonic increase of favorable genotypes with age was tested using logistic regression (see Text S1 for details).

Most of the genes we have studied, however, have not been reported to differ significantly in frequency between control subjects and centenarians—e.g., lipoprotein *lipase* (*LPL*), *lymphotoxin alpha* (*LTA*), *low-density lipoprotein receptor* (*LDLR*), and others (see Figure 1). Lack of statistically significant differences, however, do not signify the irrelevance of these genes to the aging process, since the frequency analysis of the intermediate age groups may well reveal a more complex pattern, as will be shown below.

### Identifying Buffered Age-Related Disease Genes

To identify buffered disease genes further, a more detailed examination is required. A deleterious genotype at an age-related buffered disease gene is predicted to decline initially as the population ages. As the population approaches extreme longevity, the initial decline should reverse, and the prevalence of the deleterious genotype should increase. Indeed, studies in French and Italian centenarian subjects [26–28] reported the paradox of an unfavorable genotype and phenotype that are more common in centenarians. Among the set of genes tested, we have identified two such genes: one is LPA (see Figure 3), which is associated with increased risk for vascular diseases in the elderly [29]. The other gene shown in Figure 3 is the age-related disease gene KLOTHO*,* an aging gene that is associated with low HDL and reduced CVD risk [30,31]. The reported frequency trends for both LPA and KLOTHO follow the expected *U*-shape trends based on individuals from the control group only, as described above.

Using statistical assumptions described above (see Methods and Text S1), we arrived at a statistically significant quadratic component for the genotypic frequency of LPA (*p* < 0.035). A similar statistically significant result was shown for KLOTHO (*p* < 0.035). Interestingly, LPA plasma levels, known to be a factor for coronary artery disease [32], have also been measured and found to reflect precisely the trends of the genotype: 15.3 ± 1.8 mg/dl in subjects 60–70 years old, declined to 10.8 ± 2.1 mg/dl in subjects aged 71–80 years old, with an increment to 15.7 ± 2.3 mg/dl when subjects achieve an age of 100 years old.

### Longevity Genes and Their Target Age-Related Disease Genes

To complete our analysis, the buffering effect of longevity genes on their target age-related disease genes needs to be determined. As explained above, to ensure the presence of favorable longevity genes in a younger population, we supplemented the control population with the offspring of centenarians. We then divided the pooled, control offspring population into two subpopulations—those endowed with, and those not endowed with, a favorable genotype in a longevity gene, i.e., CETP-VV versus CETP*-IV* / CETP*-II*. When a chi-square test to reveal the interaction between LPA and CETP genotypes is performed between the 92-year-old age group (advanced in age, though not yet centenarians) and the younger 80-year-old group, we find a significant interaction with respect to CETP-VV and CETP*-IV* / CETP*-II* (*p* < 0.026, chi-square). However, the hypothesis described above suggests a stronger result: in a subpopulation endowed with the favorable CETP genotype (CETP-VV), the frequency trend of the deleterious genotype of LPA will exhibit no change, or may even increase, with age. In contrast, a significant decline should be observed in the subpopulation lacking the favorable longevity CETP genotype. Indeed, such a difference in frequency trends is observed (see Figure 4). The reported differences between the observed trends is statistically significant, *p* < 0.037.

**Figure 5:**
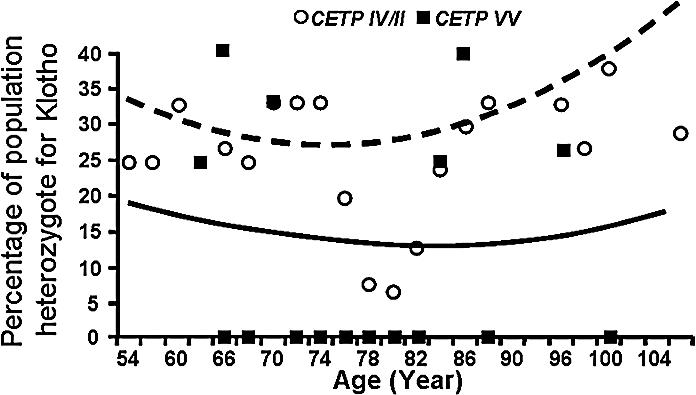
Interaction between CETP and the Buffered KLOTHO gene Comparable to Figure 4, we show here the frequency trends across ages of deleterious genotype in KLOTHO in the subpopulation having favorable longevity genotype CETP-VV versus subpopulation lacking it, i.e., CETP*-IV* and CETP*-II*. Because of the lack of offspring data, we included centenarians (see text). The two frequency trends follow a similar *U*-shape with age and show no significant interaction term (*p* < 0.38). Nonsignificant results have also been observed for the interaction term between APOC-3 and KLOTHO (unpublished data).

Although we expect to have multiple targets for each of the longevity genes, not all age-related disease genes may be targeted by all longevity genes. In case such protection is not provided, we would not therefore expect to see a significant change in frequency trends between the two subpopulations. For example, when the same pooled control offspring population was subdivided on the basis of the favorable genotype at the APOC-3 locus to test its effect on the deleterious LPA genotype, no significant changes in trend behavior were observed. In addition, as a test of our hypothesis, a similar interaction analysis was performed on the KLOTHO genotype. Since no data from centenarian's offspring were available for the gene–gene interaction analysis, we included individuals from the centenarian population. When an interaction between CETP and KLOTHO exists, one would expect the admixed, control-centenarians population to exhibit a more significant interaction term than that obtained by an admixed control-offspring population. However, no significant interaction was found when associating CETP or APOC-3 genotypes individually with the unfavorable KLOTHO genotype (P = 0.38 and P = 0.85, respectively). Figure 5 shows that the KLOTHO's frequencies for the two subpopulations, with and without favorable CETP genotype, follow a similar *U*-shape trend. The lack of interaction suggests that, though CETP and KLOTHO may both have an influence on lipoprotein size, no buffering mechanism can be inferred.

### Toward Building the Genetic Network of Aging

**Figure 6:**
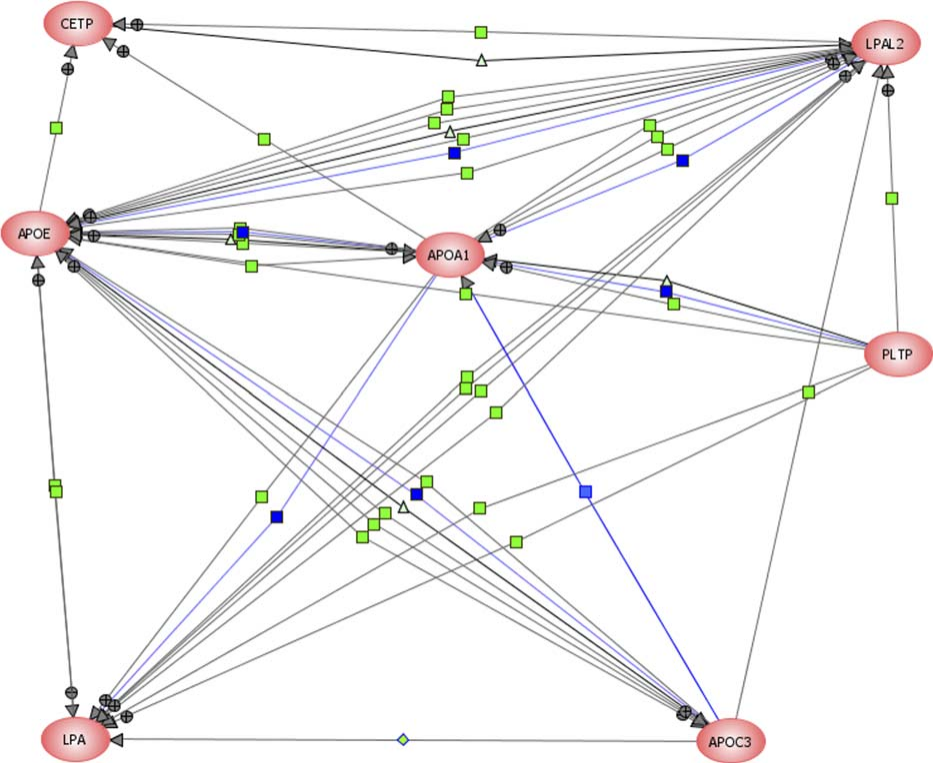
Protein–Protein Interaction Network Protein–Protein interaction networks of subsets of proteins relevant to the buffering hypothesis analysis. Connecting lines between gene symbols indicate interactions; different types of interactions are denoted by symbols on the lines. Green square, regulation; blue square, expression; light green triangle, transport; + in grey circle, positive effect; − in grey circle, negative effect. CETP interacts with LPA through LPAL2, an LPA-like 2 protein. This interaction is also mediated by LPA*L2* through *APOA1,* and apolipoprotein A-I. Also, though our analysis shows no interaction between LPA and APOC-3, pathway analysis shows a direct interaction, suggesting additional and non-overlapping functionality between CETP and APOC-3 (see text).

Finally, to corroborate our findings with existing knowledge about interactions among the set of genes we have identified, we applied a two-stage network analysis. First, using GRID (General Repository of Interaction Database), we identified all the possible targets of CETP, LPA, and APOC-3 (primary interactions), and then extended the scope to include secondary and tertiary interactions which resulted in a network of 248 nodes and 450 edges. From this extended network, we then selected those genes that lie on the minimum pathways (shortest path length linking two nodes) among CETP, LPA, and APOC-3. This resulted in the following additional genes: *APOE,* LPA*L2, PLTP,* and *APOA1*. In the second stage, we used the PathwayArchitect software application (Stratagene, http://www.strategene.com) to further corroborate the resulting interaction network. Results of this analysis yielded a similar outcome, that is, the same genes resulted in having the highest confidence index of interactions in the pathway layout graph. Figure 6 delineates a subnetwork of known interactions among proteins relevant to our hypothesis. As can be observed, CETP indeed interacts with LPA. This interaction is two-fold, via LPA*L2,* a LPA*-like 2* lipoprotein, and again, through LPA*L2* via *APOA1* apolipoprotein A-1 [33,34]. These findings call for further analysis of the role of the LPA*L2* and *APOA1* proteins. Interestingly, although the pathway analysis revealed direct contact between APOC-3 and LPA [35–38], the hypothesis test revealed that buffering does not occur between APOC-3 and LPA, since there exists no significant interaction (*p* = 0.076). This observation suggests that given the similar biological effect APOC-3 and CETP has on lipoprotein size, buffering is most likely mediated by other, yet to be discovered, biological means. Finally, when the KLOTHO protein was introduced into the pathway analysis, no additional links were found, nor did we find any link between KLOTHO and any of the subset of proteins tested. This finding is in accord with our hypothesis, due to lack of interaction between CETP or APOC-3 and KLOTHO*.* Corroborating our hypothesis with known protein interactions indicates that our analysis can predict and be used to further suggest interactions not yet known.

## Discussion

Recent progress in the search for candidate aging genes in centenarian studies has been significantly helped by the availability of more sensitive statistical techniques [39,40]. For example, an allele-specific association with longevity was found for SOD2 [41] due to the increased sensitivity of the relative risk method [42,43] over the gene frequency method [41]. Given the important polygenic aspects of the aging phenotype, an approach that searches for candidates within their genetic context is urgently needed. Previous work has already demonstrated the importance of genetic background for longevity, suggesting a role for epistasis [44,45]. The study we present here moves further in this direction by not only giving the genetic background a central role, but furthermore suggesting a novel mechanistic explanation based upon previous theoretical results on gene–gene interactions and buffering.

A previously unfavorable LPA genotype has been reported to be paradoxically increased in French and Italian studies of centenarians [26–28], representing an increased frequency of disease genotype in centenarians. Our results suggest that a harmful genotype probably does not turn into a protective one, but rather indicates a protection by other favorable longevity genotypes. Indeed we show that increased CETP-VV genotype, CETP levels, and favorable lipoprotein sizes may buffer the deleterious effects of LPA genotypes, thus allowing the accumulation of unfavorable genotypes among centenarians. Similar *U-*shaped patterns of genotypic frequency with respect to age have also been reported by Tan et al. [42] for the C282Y allele and by Cavallone et al. [46] for the PON1 gene, making them candidates for further analysis as buffered age-related disease genes.

To identify the frequency pattern of genotypes associated with aging and longevity, any analysis must include representation of all age groups. Because the nadirs of frequencies for LPA and KLOTHO genotypes were at age ∼80, previous studies that identified putative longevity genotypes (due to rising life expectancy from ages 80 to 100), may have identified protected aging genes but not true longevity genes. Comparing populations at ∼50 and ∼100 years old would potentially exclude individuals possessing aging genes that may be buffered at a later age. It is also important to realize that, because we have obtained a large number of rare individuals with exceptional longevity, classical genetic rules, such as the Hardy-Weinberg equilibrium, do not apply in this population. This should be retrospectively considered in groups of centenarians whose results were dropped because they violated the Hardy-Weinberg equilibrium. In other words, because the genotypes of survivors are “selected,” the greater the attribution of a genotype to longevity, the greater is the divergence from Hardy-Weinberg equilibrium among the elderly.

The focus of the current study has been on the longevity genes of centenarians. However, given the complexity of the trait, which may derive from multiple, redundant pathways, pleiotropic interactions, and other environmental factors, it is highly likely that different individuals achieve longevity by different means. Parallel studies in other isolated populations, such as the Icelandic, will provide the means to address new pathways for longevity. Confirming these findings in the general population will also facilitate identification of longevity genes at the individual level. It is most likely that longevity involves a far more complex relationship among longevity and disease genes than the pairwise interactions we have introduced here. Yet, our results suggest that this approach can contribute to our understanding of processes as complex as aging. We used two examples of genotypes that seemed to protect from several age-related conditions. Thus, we expect each of these genotypes to provide protection against several aging genotypes. Indeed, we conclude that investigating the genetic pathways for “aging phenotypes,” such as age-related disease and the pathways that buffer these effects, combined with analyses of quantitative traits, may suggest strategies to modulate the disease phenotypes of aging. For example, if any single longevity gene buffers against several aging genes, agents could be developed to exploit such a drug with widespread protective effects.

## Methods

### Population and genotypes studied.

Our population, Ashkenazi Jews, is an ideal study group because social, political, and religious pressures limited this population to a relatively few founders [47]. This genetic homogeneity is paralleled by a relatively homogeneous socioeconomic and educational status. Inbreeding in this population has allowed successful genetic research in Ashkenazi Jews, including the characterization of multiple rare autosomal recessive disorders such as Tay-Sachs disease [48], factor XI deficiency [49], and hyperinsulinemic hypoglycemia of infancy (i.e*.*, sulfonylurea receptor mutations) [50], as well as common diseases such as breast and ovarian cancer (i.e., *BRCA1* and *BRCA2* gene mutations) [8]. A limitation of this model is the lack of inclusion of a complex gene-environment interaction. However, by selecting an environmentally homogeneous population, we minimize the effects of such interaction.

Three hundred and five probands with exceptional longevity (228 females and 77 males, age 98.2 (0.36) years [mean (SE)], range 95–109 years; 48% over the age of 100 years) were recruited to participate in the study. Birth certificates or dates of birth as stated on passports defined the participants' ages. Probands were required to have been living independently at 95 years of age as a reflection of good health, although at the time of recruitment they could be at any level of dependency. In addition, for inclusion probands were required to have a child who was willing to participate in the study. The offspring group consisted of 227 females and 203 males (age 68.3 [0.45] years, range 54–89 years). Finally, the control group consisted of 265 females and 203 males (age 69.5 [0.52] years, range 54–90 years), matched in age to the offspring. Details regarding the recruitment and demographics of this group can be found in [11,16,17] (see Table 1 for age distribution of the subjects recruited).

**Table 1:**
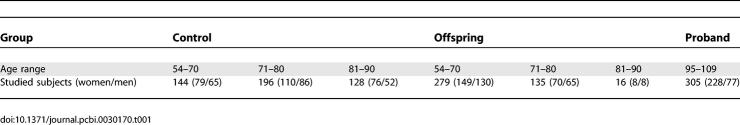
Total Number of Subjects Recruited for This Study Grouped According to Class, Age, and Gender

To test our hypothesis, we determined the prevalence of 66 common polymorphic sites in 36 genes that are known to be risk factors for cardiovascular disease (CVD) using a multilocus PCR-based genotyping assay. Briefly, DNA was amplified using multiplex reaction containing biotinylated primer pairs. Amplified fragments within each PCR product pool were then detected colorimetrically with sequence-specific oligonucleotide probes immobilized in a linear array on nylon membrane strips. Probe specificities had previously been confirmed by sequencing and by use of DNA genotyped independently through other methods such as restriction length polymorphism analysis [51].

### Statistical considerations.

The following are the statistical considerations in identifying potential buffered disease genes contributing to the aging phenotype. Among the SNPs which demonstrate a significant decline in frequency with age in the control group, we examined those in which the prevalence is significantly higher in the centenarian population relative to the control 80–90 age group. The identified SNPs are those associated with candidate-buffered disease genes. For those SNPs which show an initial significant decline with age followed by a significant increase, that is, a *U*-shape trend of age-related target genes, we confirm the pattern by fitting a generalized linear model with data from the combined control and centenarian groups. We use a binomial model with an identity link function and both linear and quadratic terms for age, and test for the significant quadratic component. More specifically, the binary response (Y) of having (Y = 1) or not having (Y = 0) the deleterious genotype of an age-related disease gene is modeled as *P*(*Y = 1*) *= b*_0_ *+ b*_1_age *+ b*_2_age^2^. Note that the standard logistic regression does not apply to this case since it models probability as monotonic to covariates. Maximum likelihood estimates of the coefficients *b*_0_ *, b*_1_ *, b*_2_ are obtained by the Fisher Scoring method. The statistical significance of the quadratic term is then determined by the likelihood ratio test that compares the likelihood of the model of b_2_ ≠ 0 with the model of b_2_ = 0. If the quadratic term is significant and the minimum of the quadratic function −*b*_1_ */*(*2b*_2_) falls within age range of our subjects, the relevant gene is further considered. To formally test the statistical significance of the interaction term between frequency trends of the two subpopulations (those endowed with and without favorable genotypes at the longevity gene), it is equivalent to test the statistical significance of interaction effects between the factor “age” and the “longevity genotype” factor, using logistic regression. The binary response in the logistic regression is whether or not a subject has the deleterious genotype of the buffered disease gene. To test the significance of age–gene interaction, the model with main effect only and the model with the interaction effects are compared using log-likelihood ratio test (for additional information see Text S1).

## Supporting Information

Figure S1 Trends of Genotypic Frequency with Age Line-a, longevity genes. Line-b, age-related diseases genes. Line-c, buffered age-related disease genes.
(11 KB PDF)

Figure S2 Trends of Genotypic Frequency with Age of Buffered Age-Related Diseases Genes Line-a, gene–gene interaction with favorable genotypes in longevity genes. Line-b, gene–gene interaction with no favorable genotypes in longevity genes.
(11 KB PDF)

Figure S3 Trend of Genotypic Frequency in Simulated Age-Structured Population (22 KB PDF)

Table S1 Genes and Their Associated SNPs Genotyped for This Study Data is represented visually in the main text (Figure 1).
(118 KB DOC)

Text S1 Supplementary Text Elaborations on analytical considerations, statistical consideration in determining longevity genes, statistical consideration in determining buffered disease genes, statistical considerations in determining interaction between longevity genes and buffered disease genes, and method and list of genes for which SNPs analysis was performed.
(39 KB DOC)

## Abbreviations

APOC-3: apolipoprotein C3
CETP: cholesteryl ester transfer protein
CVD: cardiovascular disease
LPA: *lipoprotein(a)*

## References

[pcbi-0030170-b001] Olshansky SJ, Passaro DJ, Hershow RC, Layden J, Carnes BA (2005). A potential decline in life expectancy in the United States in the 21st century. N Engl J Med.

[pcbi-0030170-b002] Kenyon C (2001). A conserved regulatory system for aging. Cell.

[pcbi-0030170-b003] Larsen PL (2001). Asking the age-old questions. Nat Genet.

[pcbi-0030170-b004] Guarente L, Kenyon C (2000). Genetic pathways that regulate ageing in model organisms. Nature.

[pcbi-0030170-b005] Nissen H, Hansen AB, Guldberg P, Petersen NE, Larsen ML (1994). Detection of a single base deletion in codon 424 of the low density lipoprotein receptor gene in a Danish family with familial hypercholesterolemia. Atherosclerosis.

[pcbi-0030170-b006] Descamps O, Hondekjin JC, Van Acker P, Deslypere JP, Heller FR (1997). High prevalence of a novel mutation in the exon 4 of the low-density lipoprotein receptor gene causing familial hypercholesterolemia in Belgium. Clin Genet.

[pcbi-0030170-b007] Yu CE, Oshima J, Wijsman EM, Nakura J, Miki T (1997). Mutations in the consensus helicase domains of the Werner syndrome gene. Werner's Syndrome Collaborative Group. Am J Hum Genet.

[pcbi-0030170-b008] Lancaster JM, Carney ME, Futreal PA (1997). BRCA 1 and 2—A genetic link to familial breast and ovarian cancer. Medscape Womens Health.

[pcbi-0030170-b009] Roth GS, Lane MA, Ingram DK, Mattison JA, Elahi D (2002). Biomarkers of caloric restriction may predict longevity in humans. Science.

[pcbi-0030170-b010] Atzmon G, Rincon M, Schechter CB, Shuldiner AR, Lipton RB (2006). Lipoprotein genotype and conserved pathway for exceptional longevity in humans. PLoS Biol.

[pcbi-0030170-b011] Barzilai N, Atzmon G, Schechter C, Schaefer EJ, Cupples AL (2003). Unique lipoprotein phenotype and genotype associated with exceptional longevity. JAMA.

[pcbi-0030170-b012] Barzilai N, Atzmon G, Derby CA, Bauman JM, Lipton RB (2006). A genotype of exceptional longevity is associated with preservation of cognitive function. Neurology.

[pcbi-0030170-b013] Atzmon G, Gabriely I, Greiner W, Davidson D, Schechter C (2002). Plasma HDL levels highly correlate with cognitive function in exceptional longevity. J Gerontol A Biol Sci Med Sci.

[pcbi-0030170-b014] Gavrilov L, Gavrilova N, Semyonova V, Evdokushkina G (2003). Early-life seasonal programming of human longevity. Proceedings of the 56th Annual Meeting of the Gerontological Society of America.

[pcbi-0030170-b015] Perls TT, Wilmoth J, Levenson R, Drinkwater M, Cohen M (2002). Life-long sustained mortality advantage of siblings of centenarians. Proc Natl Acad Sci U S A.

[pcbi-0030170-b016] Atzmon G, Schechter C, Greiner W, Davidson D, Rennert G (2004). Clinical phenotype of families with longevity. J Am Geriatr Soc.

[pcbi-0030170-b017] Barzilai N, Atzmon G, Schechter C, Lipton R, Shuldiner AR (2004). Genetic factors in exceptional longevity—Reply. JAMA.

[pcbi-0030170-b018] Terry DF (2004). Cardiovascular disease delay in centenarian offspring: Role of heat shock proteins. Ann N Y Acad Sci.

[pcbi-0030170-b019] Terry DF, McCormick M, Andersen S, Pennington J, Schoenhofen E (2004). Cardiovascular disease delay in centenarian offspring. J Gerontol A Biol Sci Med Sci.

[pcbi-0030170-b020] Terry DF, Wilcox M, McCormich MA, Lawler E, Perls TT (2003). Cardiovascular advantages among the offspring of centenarians. J Gerontol A Biol Sci Med Sci.

[pcbi-0030170-b021] Rutherford SL, Lindquist S (1998). Hsp90 as a capacitor for morphological evolution. Nature.

[pcbi-0030170-b022] Queitsch C, Sangster TA, Lindquist S (2002). Hsp90 as a capacitor of phenotypic variation. Nature.

[pcbi-0030170-b023] Bergman A, Siegal ML (2003). Evolutionary capacitance as a general feature of complex gene networks. Nature.

[pcbi-0030170-b024] Siegal ML, Bergman A (2002). Waddington's canalization revisited: Developmental stability and evolution. Proc Natl Acad Sci U S A.

[pcbi-0030170-b025] Groenendijk M, Cantor RM, de Bruin TW, Dallinga-Thie GM (2001). The apoAI-CIII-AIV gene cluster. Atherosclerosis.

[pcbi-0030170-b026] Malaguarnera M, Giugno I, Ruello P, Rizzo M, Panebianco MP (1998). Lipid profile variations in a group of healthy elderly and centenarians. Eur Rev Med Pharmacol Sci.

[pcbi-0030170-b027] Pepe G, Di Perna V, Resta F, Lovecchio M, Chimienti G (1998). In search of a biological pattern for human longevity: Impact of apo A-IV genetic polymorphisms on lipoproteins and the hyper-Lp(a) in centenarians. Atherosclerosis.

[pcbi-0030170-b028] Thillet J, Doucet C, Chapman J, Herbeth B, Cohen D (1998). Elevated lipoprotein(a) levels and small apo(a) isoforms are compatible with longevity: Evidence from a large population of French centenarians. Atherosclerosis.

[pcbi-0030170-b029] Kovesdy CP (2004). Lp(a) lipoprotein, vascular disease, and mortality in the elderly. N Engl J Med.

[pcbi-0030170-b030] Arking DE, Becker DM, Yanek LR, Fallin D, Judge DP (2003). KLOTHO allele status and the risk of early-onset occult coronary artery disease. Am J Hum Genet.

[pcbi-0030170-b031] Arking DE, Atzmon G, Arking A, Barzilai N, Dietz HC (2005). Association between the functional variant of KLOTHO allele and high-density lipoprotein cholesterol, blood pressure, stroke, and longevity. Circ Res.

[pcbi-0030170-b032] Anuurad E, Boffa MB, Koschinsky ML, Berglund L (2006). Lipoprotein(a): A unique risk factor for cardiovascular disease. Clin Lab Med.

[pcbi-0030170-b033] Escola-Gil JC, Julve J, Marzal-Casacuberta A, Ordóñez-Llanos J, González-Sastre F (2001). ApoA-II expression in CETP transgenic mice increases VLDL production and impairs VLDL clearance. J Lipid Res.

[pcbi-0030170-b034] Pussinen PJ, Jauhiainen, Metso J, Pyle LE, Marcel YL (1998). Binding of phospholipid transfer protein (PLTP) to apolipoproteins A-I and A-II: Location of a PLTP binding domain in the amino terminal region of apoA-I. J Lipid Res.

[pcbi-0030170-b035] Jones DR, Leffak M (1999). A bifunctional regulatory element of the human ApoA-I gene responsive to a distal enhancer. DNA Cell Biol.

[pcbi-0030170-b036] Kostner KM, Kostner GM (2004). Factors affecting plasma lipoprotein(a) levels: Role of hormones and other nongenetic factors. Semin Vasc Med.

[pcbi-0030170-b037] Walsh A, Azrolan N, Wang K, Marcigliano A, O'Connell A (1993). Intestinal expression of the human apoA-I gene in transgenic mice is controlled by a DNA region 3′ to the gene in the promoter of the adjacent convergently transcribed apoC-III gene. J Lipid Res.

[pcbi-0030170-b038] Zannis VI, Kan HY, Kritis A, Zanni E, Kardassis D (2001). Transcriptional regulation of the human apolipoprotein genes. Front Biosci.

[pcbi-0030170-b039] Yashin AI, De Benedictis G, Vaupel JW, Tan Q, Andreev KF (1999). Genes, demography, and life span: The contribution of demographic data in genetic studies on aging and longevity. Am J Hum Genet.

[pcbi-0030170-b040] Yashin AI, De Benedictis G, Vaupel JW, Tan Q, Andreev KF (2000). Genes and longevity: Lessons from studies of centenarians. J Gerontol A Biol Sci Med Sci.

[pcbi-0030170-b041] De Benedictis G, Tan Q, Jeune B, Christensen K, Ukraintseva SV (2001). Recent advances in human gene-longevity association studies. Mech Ageing Dev.

[pcbi-0030170-b042] Tan Q, Bathum L, Christiansen L, De Benedictis G, Bellizzi D (2003). Logistic regression models for polymorphic and antagonistic pleiotropic gene action on human aging and longevity. Ann Hum Genet.

[pcbi-0030170-b043] Tan Q, De Benedictis G, Yashi AI, Bonafe M, DeLuca M (2001). Measuring the genetic influence in modulating the human life span: Gene–environment interaction and the sex-specific genetic effect. Biogerontology.

[pcbi-0030170-b044] Spencer CC, Howell CE, Wright AR, Promislow DEL (2003). Testing an “aging gene” in long-lived *Drosophila* strains: Increased longevity depends on sex and genetic background. Aging Cell.

[pcbi-0030170-b045] Spencer CC, Promislow DE (2005). Age-specific changes in epistatic effects on mortality rate in Drosophila melanogaster. J Hered.

[pcbi-0030170-b046] Cavallone L, Bonafe M, Olivieri F, Cardelli M, Marchegiani F (2003). The role of IL-1 gene cluster in longevity: A study in Italian population. Mech Ageing Dev.

[pcbi-0030170-b047] Eisenstein B, Stark H, Goodman RM (1979). Benign familial haematuria in children from the Jewish communities of Israel: Clinical and genetic studies. J Med Genet.

[pcbi-0030170-b048] Peleg L, Karpati M, Gazit E, Raas-Rothschild A, Goldman B (1994). Mutations of the hexosaminidase A gene in Ashkenazi and non-Ashkenazi Jews. Biochem Med Metab Biol.

[pcbi-0030170-b049] Shpilberg O, Peretz H, Zivelin A, Yatuv R, Chetrit A (1995). One of the two common mutations causing factor XI deficiency in Ashkenazi Jews (type II) is also prevalent in Iraqi Jews, who represent the ancient gene pool of Jews. Blood.

[pcbi-0030170-b050] Nestorowicz A, Wilson BA, Schoor KP, Inoue H, Glaser B (1996). Mutations in the sulonylurea receptor gene are associated with familial hyperinsulinism in Ashkenazi Jews. Hum Mol Genet.

[pcbi-0030170-b051] Cheng S, Grow MA, Pallaud C, Klitz W, Erlich HA (1999). A multilocus genotyping assay for candidate markers of cardiovascular disease risk. Genome Res.

