# Supplementary material for: Buffering Mechanisms in Aging: A Systems Approach Toward Uncovering the Genetic Component of Aging
Source: PLoS Comput Biol. 2007 Aug 31;3(8):e170. doi: 10.1371/journal.pcbi.0030170 (PMC1963511; doi:10.1371/journal.pcbi.0030170)
Supplement: Figure S1 — Line-a, longevity genes. Line-b, age-related diseases genes. Line-c, buffered age-related disease genes. (11 KB PDF) [file pcbi.0030170.sg001.pdf]

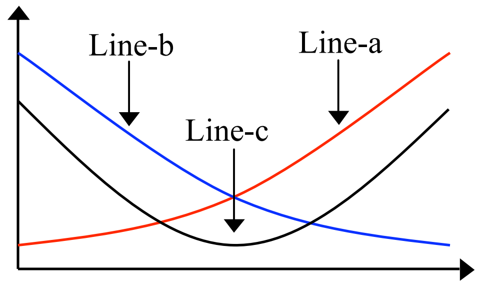

Figure S1: Trends of Genotypic Frequency with Age.
Line-a - longevity genes; Line-b - age related diseases genes;
Line-c - buffered age related disease genes.
