# Supplementary material for: Buffering Mechanisms in Aging: A Systems Approach Toward Uncovering the Genetic Component of Aging
Source: PLoS Comput Biol. 2007 Aug 31;3(8):e170. doi: 10.1371/journal.pcbi.0030170 (PMC1963511; doi:10.1371/journal.pcbi.0030170)
Supplement: Figure S2 — Line-a, gene–gene interaction with favorable genotypes in longevity genes. Line-b, gene–gene interaction with no favorable genotypes in longevity genes. (11 KB PDF) [file pcbi.0030170.sg002.pdf]

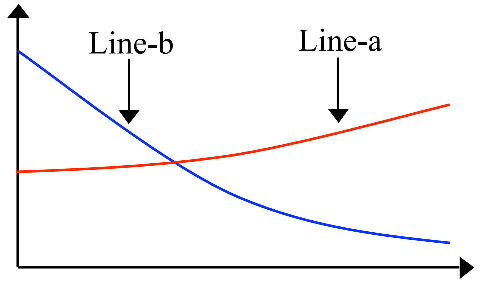

Figure S2: Trends of Genotypic Frequency with Age of Buffered Age-Related Diseases Genes. Line-a - gene-gene interaction with favorable genotypes in longevity genes. Line-b - gene-gene interaction with no favorable genotypes in longevity genes.
