# Supplementary figures and images for: Buffering Mechanisms in Aging: A Systems Approach Toward Uncovering the Genetic Component of Aging
Source: PLoS Comput Biol. 2007 Aug 31;3(8):e170. doi: 10.1371/journal.pcbi.0030170 (PMC1963511; doi:10.1371/journal.pcbi.0030170)

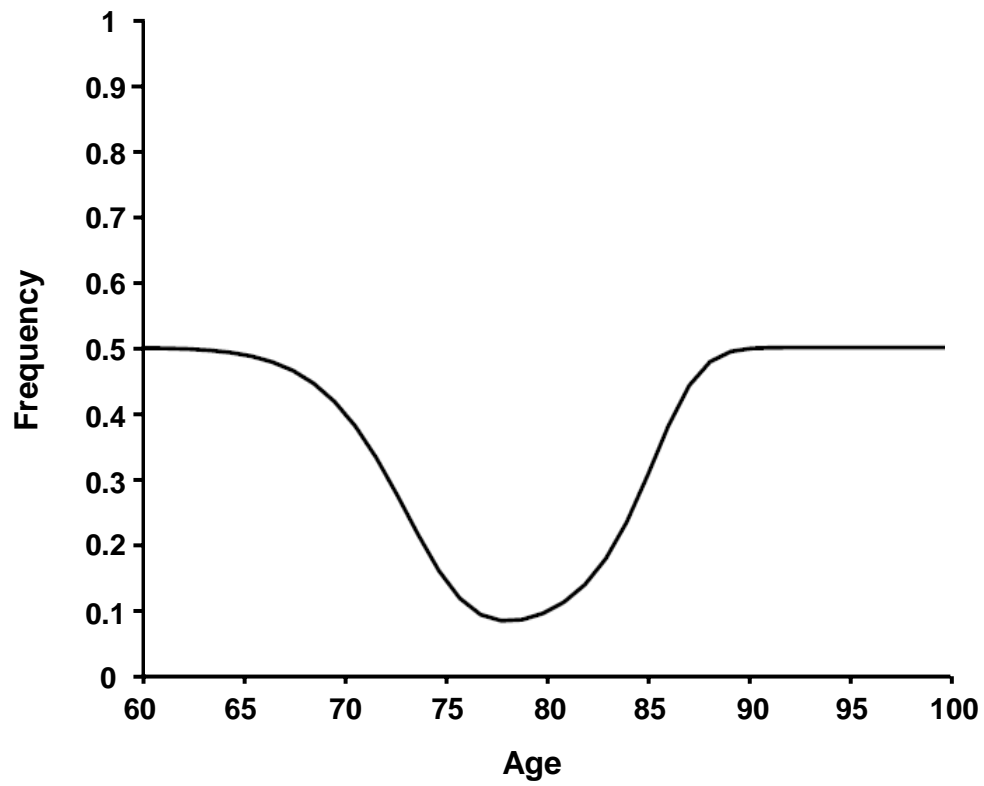

**FIGURE 3S: Trend of Genotypic Frequency in simulated Age-structured population**

Supplement: Figure S3 — (22 KB PDF) [file pcbi.0030170.sg003.pdf]
