# Supplementary material for: Buffering Mechanisms in Aging: A Systems Approach Toward Uncovering the Genetic Component of Aging
Source: PLoS Comput Biol. 2007 Aug 31;3(8):e170. doi: 10.1371/journal.pcbi.0030170 (PMC1963511; doi:10.1371/journal.pcbi.0030170)
Supplement: Table S1 — Data is represented visually in the main text (Figure 1). (118 KB DOC) [file pcbi.0030170.st001.doc]

**Table S1.** Genes and their associated SNPs genotyped for this study. Data is represented visually in the main text (Figure 1).

| **Gene symbol** | **Gene name** | **Polymorphism** | **Location** | **Frequency^*^** |
| --- | --- | --- | --- | --- |
| ACE (DCP1) | angiotensin I-converting enzyme | intron 16 ins/del | 17q23 | 58/43 (0.01) |
| ADD1 | adducin 1; alpha adducin | gly460trp (G/T) | 4p16.3 | 75/74 (0.768) |
| ADRB2 | beta-2-adrenergic receptor | arg16gly (A/G) | 5q32 | 16/21 (0.188) |
|  |  | gln27glu (C/G) | 5q32 | 31/40 (0.04) |
| ADRB3 | beta-3-adrenergic receptor | trp64arg (T/C) | 8p11 | 97/92 (0.01) |
| AGT | angiotensin I | met235thr (T/C) | 1q42 | 41/33 (0.057) |
| AGTR1 | angiotensin II receptor, type 1 | 1166 A/C | 3q24 | 45/48 (0.56) |
| APOA4 | apolipoprotein A-IV | thr347ser (A/T) | 11q23.3 | 70/69 (0.81) |
|  |  | gln360his (G/T) | 11q23.3 | 1/0.5 (0.18) |
| APOB | apolipoprotein B | thr71ile (C/T) | 2p24 | 80/71 (0.03) |
|  |  | arg3500gln (G/A) | 2p24 | 97/100 (0.01) |
| APOC3 | apolipoprotein C-III | -641 C/A | 11q23.3 | 10/25 (0.0001) ^**^ |
|  |  | -482 C/T | 11q23.3 | 61/54 (0.08) |
|  |  | -455 T/C | 11q23.3 | 28/33 (0.18) |
|  |  | 1100 C/T | 11q23.3 | 63/51 (0.007) |
|  |  | 3175 C/G | 11q23.3 | 80/77 (0.35) |
|  |  | 3206 T/G | 11q23.3 | 37/38 (0.84) |
| APOE | apolipoprotein E | cys112arg (T/C) | 19q13 | 82/88 (0.035) |
|  |  | arg158cys (C/T) | 19q13 | 91/84 (0.02) |
| CBS | cystathionine beta-synthase | ile278thr, 68-bp ins | 21q22.3 | 100/100 (1) |
| CETP | cholesteryl ester transfer protein | -631 C/A | 16q21 | 47/34 (0.002) |
|  |  | -629 C/A | 16q21 | 100/87 (0.01) |
|  |  | ile405val (A/G) | 16q21 | 8/23 (0.0001) ^**^ |
|  |  | asp442gly (A/G) | 16q21 | 98/100 (0.12) |
|  |  | intron 14 G(+1)A | 16q21 | 99/100 (0.13) |
|  |  | intron 14 (+3)Tins | 16q21 | 100/100 (1) |
|  |  | intron 1 TaqIB +/- (G/A) | 16q21 | 38/40 (0.7) |
| F2 | coagulation factor II or prothrombin | 20210 G/A | 11p11.1 | 91/94 (0.246) |
| F5 | coagulation factor V | arg506gln (G/A) | 1q23 | 94/95 (0.775) |
| F7 | coagulation factor VII | -323 10-bp del/ins | 13q34 | 3/5 (0.24) |
|  |  | arg353gln (G/A) | 13q34 | 71/67 (0.489) |
| FGB | fibrinogen, beta polypeptide chain | -455 G/A | 4q28 | 59/53 (0.183) |
| GNB3 | guanine nucleotide-binding protein, beta-3 | 825 C/T | 12p13 | 39/50 (0.01) |
| ICAM1 | intercellular adhesion molecule 1 | gly214arg (G/A) | 19p13 | 96/96 (0.658) |
| ITGA2 | integrin, alpha-2 | 873 G/A | 5q11.2 | 39/40 (0.728) |
| ITGB3 | integrin, beta-3 | leu33pro (T/C) | 17q21 | 70/71 (0.73) |
| LDLR | low density lipoprotein receptor | NcoI+/- (A/G) | 19p13.2 | 50/56 (0.19) |
| LIPC | lipase, hepatic | -480 C/T | 15q21 | 3/4 (0.66) |
| LPA | lipoprotein, Lp(a) | 93 C/T | 6q26 | 0.8/1 (0.82) |
|  |  | 121 G/A | 6q26 | 2/4 (0.33) |
| LPL | lipoprotein lipase | -93 T/G | 8p22 | 95/95 (0.51) |
|  |  | asp9asn (G/A) | 8p22 | 97/98 (0.27) |
|  |  | asn291ser (A/G) | 8p22 | 96/97 (0.42) |
|  |  | ser447term (C/G) | 8p22 | 0.6/0.4 (0.75) |
| LTA | lymphotoxin alpha | thr26asn (C/A) | 6p21.3 | 66/64 (0.54) |
| MMP3 | matrix metalloproteinase 3 | -1171 A5/A6 | 11q22 | 45/44 (0.908) |
| MTHFR | 5,10-methylenetetrahydrofolate reductase | 677 C/T | 1p36 | 45/30 (0.0009) |
| NOS3 | nitric oxide synthase 3 (endothelial) | -922 A/G | 7q36 | 62/44 (0.00095) |
|  |  | -690 C/T | 7q36 | 71/86 (0.002) |
|  |  | glu298asp (G/T) | 7q36 | 65/71 (0.214) |
| NPPA | natriuretic peptide precursor A | 664 G/A | 1p36.2 | 94/94 (0.851) |
|  |  | 2238 T/C | 1p36.2 | 80/75 (0.237) |
| PAI1 (SERPINE1) | plasminogen activator inhibitor 1 | -675 G5/G4 | 7q22 | 25/27 (0.613) |
|  |  | 11053 G/T | 7q22 | 30/31 (0.924) |
| PON1 | paraoxonase 1 | met55leu (A/T) | 7q21 | 17/23 (0.17) |
|  |  | gln192arg (A/G) | 7q21 | 37/41 (0.4) |
| PON2 | paraoxonase 2 | ser311cys (C/G) | 7q21 | 2/3 (0.61) |
| PPARG | peroxisome proliferator-activated receptor-γ | pro12ala (C/G) | 3p25 | 93/88 (0.1) |
| SCNN1A | sodium channel, nonvoltage-gated 1 alpha | trp493arg (T/C) | 12p13 | 81/94 (0.0012) |
|  |  | ala663thr (G/A) | 12p13 | 76/63 (0.0015) |
| SELE | selectin E | ser128arg (A/C) | 1q23 | 72/74 (0.779) |
|  |  | leu554phe (C/T) | 1q23 | 84/88 (0.266) |
| TNF | tumor necrosis factor | -376 G/A | 6p21.3 | 95/97 (0.181) |
|  | (TNF superfamily, member 2) | -308 G/A | 6p21.3 | 88/85 (0.335) |
|  |  | -244 G/A | 6p21.3 | 91/83 (0.163) |
|  |  | -238 G/A | 6p21.3 | 92/86 (0.195) |

^*^ The reported values are percent in Control/Proband and its significance of the change (*P*-Value)

^**^ After Bonferroni correction Only *CETP-VV* and *APOC-3 CC* remain significant (*P*<0.0066).
