# Supplementary material for: Buffering Mechanisms in Aging: A Systems Approach Toward Uncovering the Genetic Component of Aging
Source: PLoS Comput Biol. 2007 Aug 31;3(8):e170. doi: 10.1371/journal.pcbi.0030170 (PMC1963511; doi:10.1371/journal.pcbi.0030170)
Supplement: Text S1 — Elaborations on analytical considerations, statistical consideration in determining longevity genes, statistical consideration in determining buffered disease genes, statistical considerations in determining interaction between longevity genes and buffered disease genes, and method and list of genes for which SNPs analysis was performed. (39 KB DOC) [file pcbi.0030170.sd001.doc]

**Supplementary Text**

**Elaborations on Analytical Considerations**

Aging is associated with a decline in the frequency of survivors into older ages, such that the frequency of centenarians in human populations declines to ~1/10,000 persons. Thus, we expect the prevalence of favorable genotypes in genetic hallmark genes which contribute to extreme longevity to be significantly lower, *or not present*, in a younger control population relative to their prevalence in centenarians. Our method of identifying the relevant genes and their potential target age-related disease genes is based on the following considerations. **(I)** The relative prevalence of favorable ‘longevity’ genotypes in the population increases as the population ages. Indeed, as mortality weeds out those individuals lacking the favorable genotype in a longevity gene, the proportion of those endowed with the favorable genotypes will increase, **Figure S1 (Line-a): longevity genes -genes contributing to longevity and protection against some age-related diseases**. **(II)** The relative prevalence of deleterious genotypes in genes associated with age-related diseases decreases as the population ages, since mortality reduces the proportion of individuals carrying the deleterious genotype, **Figure S1 (Line-b): disease genes -genes associated with age-related diseases that cannot be buffered by longevity genes**. **(III)** The relative frequency of deleterious genotypes in some disease genes decrease only within younger age cohorts, but return to near-initial frequency, or higher, as some longevity genes buffer their deleterious effect, **Figure S1 (Line-c): buffered disease genes -genes associated with age related diseases whose phenotype can be suppressed by longevity genes.**

The seemingly different processes (considerations **(I)** and **(II))**, however, cannot be disentangled, since an increase of a favorable genotype in a longevity gene necessarily results in a decrease of the frequency of its complementary unfavorable genotype. Similarly a decrease in a deleterious genotype at an age-related disease gene necessarily implies an increase in its non-deleterious alternative. In addition, our analysis cannot stop here, as the association between a specific longevity gene and its potential target buffered disease genes requires further consideration. To reveal this association, we subdivide a control population on the basis of the presence or absence of a favorable genotype in a candidate longevity gene and assay the frequency trend of a candidate buffered disease gene. The following differences between our subject groups are expected. **(I)** In the presence of favorable longevity genotypes, the frequency of a deleterious genotype in a buffered disease gene will remain unchanged with age, because its disease-inducing effect is buffered, **Figure S2 (Line-a): gene-gene interaction with favorable genotypes in longevity genes**. **(II)** In contrast, in the absence of favorable longevity genotype, the frequency of deleterious genotypes in buffered disease genes will behave like a disease gene, as its effects are not buffered by longevity gene, **Figure S2 (Line-b): gene-gene interaction with no favorable genotypes in longevity genes**.

To formally understand why we observe a *U*-shaped curve, consider the following Age-structured population model:

Individuals in the population are born into one of three categories:

1. Unprotected with age-related disease allele,
2. No age-related disease allele,
3. Protected, with or without age-related disease allele (longevity gene)

We denote the total number of individuals in each of these categories at time *t*, as *U(t)*, *N(t)*, and *P(t)* respectively. We assume the populations at time *t*=0 in each category to be constant (*U*(0)=*c_U_T*, *N*(0)=*c_N_T*, and *P*(0)=*c_P_T*, where *c_U_*=0.45, *c_N_*,=0.45 and *c_P_*=0.1 represent the relative proportions of each category within the total initial population *T*). We also assume half of the protected population (*P*) has the age-related disease allele. The age-at-death distribution is assumed Gaussian with mean values *d_U_*,=75, *d_N_*=85, and *d_P_*=100 respectively for each category. Therefore, the probability of survival *S_t_* at each time *t* is simply the value from the corresponding Gaussian CDF. We define a matrix A in which the off-diagonal elements A_t+1,t_=*S_t_*, and are zero otherwise. Simulations proceed via iterative matrix multiplication, e.g. *U_n_*_+1_=*AU_n_* up to *n*=1000 iterations to obtain a stationary distribution for each population (as far as *t*=120). We then calculate the frequency of age-related disease allele in the total population over time as (*U*(*t*)+0.5 *P*(*t*)) / (*U*(*t*)+*N*(*t*)+*P*(*t*)). The result is shown below:

**Statistical consideration in determining longevity genes.**

A logistic regression model is used to examine the monotonic increase of the favorable genotypes of APOC-3 and CETP in the pooled population of control (320) and centenarians (256). The binary response is whether the person has the favorable genotype (CC of APOC-3, VV of CETP). The covariant is the age at recruitment, and only its linear term is included in the model. The *P*-value is based on the likelihood ratio test and Chi-square approximation. The alternative hypothesis is two-sided.

**Statistical consideration in determining buffered disease genes.**

To identify buffered disease genes, we first performed Mantel’s test for trend and logistic regression, to test for a monotonic decline in genotype frequencies with age in the extended control group. For those SNPs exhibiting a significant initial decline followed by a significant increase (i.e., a *U*-shaped trend of age-related target genes), we confirm the pattern using a generalized linear model on the entire control group and the centenarian group. We use a binomial model with identity link function with both linear and quadratic terms of age and test for the significant quadratic component. More specifically, the binary response (Y) of having (Y=1) or not having (Y=0) deleterious genotype at age-related disease gene is modeled as *P(Y=1)=b_0_+b_1_*age*+b_2_*age*^2^*. Note that the standard logistic regression does not apply to this case since it models probability as monotonic to covariates. Maximum likelihood estimates of the coefficients *b_0_, b_1_, b_2_* are obtained by the Fisher Scoring method. The statistical significance of the quadratic term is determined by the likelihood ratio test that compares the likelihood of the model of b_2_≠0 with the model of b_2_=0. If the quadratic term is significant and the minimum of the quadratic function -*b_1_/(2b_2_)* falls within age range of our subjects, we take the gene as our target gene.

**Statistical considerations in determining interaction between longevity genes and buffered disease genes**

A pooled population of offspring and control individuals is divided into two subpopulations based upon presence or absence of the favorable genotype in a longevity gene. The frequency of the deleterious genotype in each of the buffered disease genes is then measured for all age groups in the pooled control-offspring sub-population. In the subpopulation having no favorable genotype at the longevity gene, a decline is expected in the frequency of the deleterious age-related disease genotype due to mortality. However, no significant change or even an increase is expected in that same deleterious age-related disease genotype in the subpopulation endowed with the favorable genotype in the longevity gene. To formally test the statistical significance of the different patterns between the favorable and the unfavorable genotypes at the longevity gene, it is equivalent to test the statistical significance of interaction effects between the factor **age** and the **longevity gene** factor *using logistic regression.* The binary response in the logistic regression is whether or not a subject has the deleterious genotype of the buffered disease gene. To test the significance of age-gene interaction, the model with main effect only and the model with the interaction effects are compared using log-likelihood ratio test. Pairs found to have significant interaction terms are further explored. Specifically, we use logistic regression to see if there is a decline in one group and no decline in the other.

**Method and List of genes for which SNPs analysis was performed.**

A multilocus polymerase chain reaction (PCR)-based assay was utilized to genotype known polymorphisms. Briefly, DNA was amplified using multiplex reaction containing biotinylated primer pairs. Amplified fragments within each PCR product pool were then detected colorimetrically with sequence-specific oligonucleotide probes immobilized in a linear array on nylon membrane stripes. Probe specificities had previously been confirmed by sequencing and by use of DNA genotyped independently through other methods such as restriction length polymorphism analysis.
